# 1-year survival in haemophagocytic lymphohistiocytosis: a nationwide cohort study from England 2003–2018

**DOI:** 10.1186/s13045-023-01434-4

**Published:** 2023-05-26

**Authors:** Joe West, Peter Stilwell, Hanhua Liu, Lu Ban, Mary Bythell, Tim Card, Peter Lanyon, Vasanta Nanduri, Judith Rankin, Mark Bishton, Colin Crooks

**Affiliations:** 1 University of Nottingham, Nottingham, UK; 2 National Disease Registration Service, NHS Digital, Leeds, UK; 3 Nottingham University Hospitals NHS Trust, Nottingham, UK; 4 PPD, Beijing, China; 5 Watford General Hospital NHS Trust, Watford, UK; 6 Newcastle University, Newcastle upon Tyne, UK; 7 Aarhus University, Aarhus, Denmark

**Keywords:** HLH, Survival, Blood cancers

## Abstract

Haemophagocytic lymphohistiocytosis (HLH) is a lethal syndrome of excessive immune activation. We undertook a nationwide study in England of all cases of HLH diagnosed between 2003 and 2018, using linked electronic health data from hospital admissions and death certification. We modelled interactions between demographics and comorbidities and estimated one-year survival by calendar year, age group, gender and comorbidity (haematological malignancy, auto-immune, other malignancy) using Cox regression. There were 1628 people with HLH identified. Overall, crude one-year survival was 50% (95% Confidence interval 48–53%) which varied substantially with age (0–4: 61%; 5–14: 76%; 15–54: 61%; > 55: 24% *p* < 0.01), sex (males, 46%, worse than females, 55% *p* < 0.01) and associated comorbidity (auto-immune, 69%, haematological malignancy 28%, any other malignancy, 37% *p* < 0.01). Those aged < 54 years had a threefold increased risk of death at 1-year amongst HLH associated with malignancy compared to auto-immune. However, predicted 1-year survival decreased markedly with age in those with auto-immune (age 0–14, 84%; 15–54, 73%; > 55, 27%) such that among those > 55 years, survival was as poor as for patients with haematological malignancy. One-year survival following a diagnosis of HLH varies considerably by age, gender and associated comorbidity. Survival was better in those with auto-immune diseases among the young and middle age groups compared to those with an underlying malignancy, whereas in older age groups survival was uniformly poor regardless of the underlying disease process.

**Supplementary Information:**

The online version contains supplementary material available at 10.1186/s13045-023-01434-4.

To the Editor:

Haemophagocytic lymphohistiocytosis (HLH) is a life-threatening clinical syndrome of excessive immune activation [1]. Multiple predisposing factors influence the likelihood of developing HLH; children frequently have inherited defects in cytotoxic lymphocyte function, younger adults have viral infections and/or autoimmunity whilst older adults most likely have underlying malignancy. Therapies are directed towards both acute inflammation and underlying trigger factors. The incidence of HLH has been increasing [2, 3] and high mortality rates, particularly those with underlying malignancy, well established [1, 4]. Published data are derived from single or a small number of large academic centres [1, 5], or specialty-centred populations such as paediatrics [1, 6, 7] over extended time-periods. We examined 1-year survival of HLH in England for all cases diagnosed 2003–2018 and modelled interactions between demographics and comorbidities with survival.

Linked electronic health records from English Hospital Episode Statistics (HES) [8], the National Cancer Registration Dataset (NCRD) [9] and Office for National Statistics (ONS) death certification data were used. Patients diagnosed with HLH were identified using our validated approach [2, 3, 10]. They included patients of all ages admitted to hospital or died between 1 January 2003 and 31 December 2018. Date of diagnosis was the first day of the admission in which HLH was coded. The presence/absence of comorbidities was identified from available HES and NCRD records prior to diagnosis of HLH and up to three months after. Where there was overlap of non-infectious comorbidities, patients were classified with a mutually exclusive hierarchy: haematological malignancy, rheumatological disease/inflammatory bowel diseases (IBD), non-haematological malignancy and none recorded. As there was no access to serological or genetic tests, the type (acute, chronic, reactivation) of viral illness nor genetic cause could not be ascertained. The number at risk from the date of HLH diagnosis and 1-year mortality frequencies were calculated by age, gender, calendar period and comorbidity.

A Cox regression model was fitted, adjusted by age, sex, comorbidities and calendar time period to assess if observed differences in survival were confounded. We fitted interactions between age and calendar year, and between comorbidity and calendar year, and tested them with general likelihood ratio tests. We used R (version 4.1.2) [11] for statistical analyses (see Additional file 1: Methods).

A total of 1628 patients were identified (Additional file 2: Fig. S1). Characteristics of the cases and 1-year survival are described in Table 1. Of the whole cohort, 461 (28.3%) had a recorded haematological malignancy, 378 (23.2%) a non-malignant comorbidity (rheumatological disease/IBD), and 107 (6.6%) a non-haematological malignancy. Overall, crude 1-year survival was 50% (95% CI 48–53%) which varied with age (0–4, 61%; 5–14, 76%; 15–54, 61%; > 55, 24% *p* < 0.01), gender (males, 46%, females, 55% *p* < 0.01) (Additional file 3: Fig. S2) and comorbidity (rheumatological/IBD, 69%, haematological, 28%, or other malignancy, 37% *p* < 0.01) (Fig. 1). Most deaths occurred within two months of diagnosis. Those aged 0–14 and 15–54 years had a threefold increased risk of death at 1-year among HLH associated with haematological or non-haematological malignancy versus rheumatological disease/IBD (Additional file 4: Fig. S3, Additional file 5: Table S1). Outcomes did not depend upon underlying cancer subtype, when split into B-cell, T-cell and Hodgkin lymphoma sub-groups or by solid organ malignancy subtype (Additional file 6: Fig. S4, Additional file 7: Fig. S5). In the adjusted model, no change in survival was observed over time.

**Table 1:** Characteristics of the HLH cohort and 1-year survival (95% CI)

Characteristic		Number at risk at start of study (%)	Number of deaths	Crude survival probability at 1 year	CI (95%)^a^
Overall	–	1628 (100.00%)	811	0.50	(0.48–0.53)
Gender	Female	708 (43.49%)	317	0.55	(0.52–0.59)
	Male	920 (56.51%)	494	0.46	(0.43–0.50)
Age group	0–4	315 (19.35%)	124	0.61	(0.55–0.66)
	5–14	196 (12.04%)	47	0.76	(0.70–0.82)
	15–34	290 (17.81%)	80	0.72	(0.67–0.78)
	35–54	266 (16.34%)	136	0.49	(0.43–0.55)
	55–74	421 (25.86%)	315	0.25	(0.21–0.30)
	75+	140 (8.60%)	109	0.22	(0.16–0.30)
Epoch	2003–2008	306 (18.80%)	134	0.56	(0.51–0.62)
	2009–2013	488 (29.98%)	235	0.52	(0.48–0.56)
	2014–2018	834 (51.23%)	442	0.47	(0.44–0.51)
Chronic conditions	Any inflammatory rheumatological disease/IBD	378 (23.22%)	140	0.63	(0.58–0.68)
	Inflammatory bowel disease	70 (4.30%)	34	0.51	(0.41–0.65)
	Adult-onset Still's disease	30 (1.84%)	7	0.77	(0.63–0.93)
	Systemic juvenile idiopathic arthritis	78 (4.79%)	8	0.90	(0.83–0.97)
	Rheumatoid arthritis	40 (2.46%)	26	0.35	(0.23–0.53)
	Other inflammatory arthritis	9 (0.55%)	5	0.44	(0.21–0.92)
	Vasculitis	64 (3.93%)	38	0.41	(0.30–0.55)
	Other connective tissue diseases	40 (2.46%)	12	0.70	(0.57–0.86)
	Systemic lupus erythematosus	47 (2.89%)	10	0.79	(0.68–0.91)
Haematological malignancies	Any haematological malignancy	461 (28.32%)	331	0.28	(0.24–0.33)
	B-cell lymphoma	114 (7.00%)	86	0.25	(0.18–0.34)
	Hodgkin lymphoma	40 (2.46%)	26	0.35	(0.23–0.53)
	Lymphoma NOS	32 (1.97%)	25	0.22	(0.11–0.42)
	T-cell lymphoma	84 (5.16%)	66	0.21	(0.14–0.32)
	Leukaemia	125 (7.68%)	94	0.25	(0.18–0.34)
	Other haematological histiocytic/myelodysplastic/malignancy/unspecified	66 (4.05%)	34	0.48	(0.38–0.62)
Non-haematological malignancies	Any non-haematological malignancy excluding non-melanoma skin cancer	107 (6.57%)	74	0.31	(0.23–0.41)
	Malignant neoplasms of breast	17 (1.04%)	10	0.41	(0.23–0.73)
	Malignant neoplasms of genital organs	21 (1.29%)	14	0.33	(0.18–0.61)
	Malignant neoplasms of urinary tract	22 (1.35%)	16	0.27	(0.14–0.54)
	Other non-haematological malignancies	47 (2.89%)	34	0.28	(0.17–0.44)
Hierarchical chronic conditions^b^	Haematological malignancy	461 (28.32%)	331	0.28	(0.24–0.33)
	Rheumatological disease or IBD	322 (19.78%)	99	0.69	(0.64–0.74)
	Non-haematological malignancy excluding non-melanoma skin cancer	72 (4.42%)	45	0.37	(0.28–0.51)
	None recorded	773 (47.48%)	336	0.57	(0.53–0.60)

^a^95% Confidence interval

^b^Deduplicated hierarchically according to order in table

**Fig. 1:**
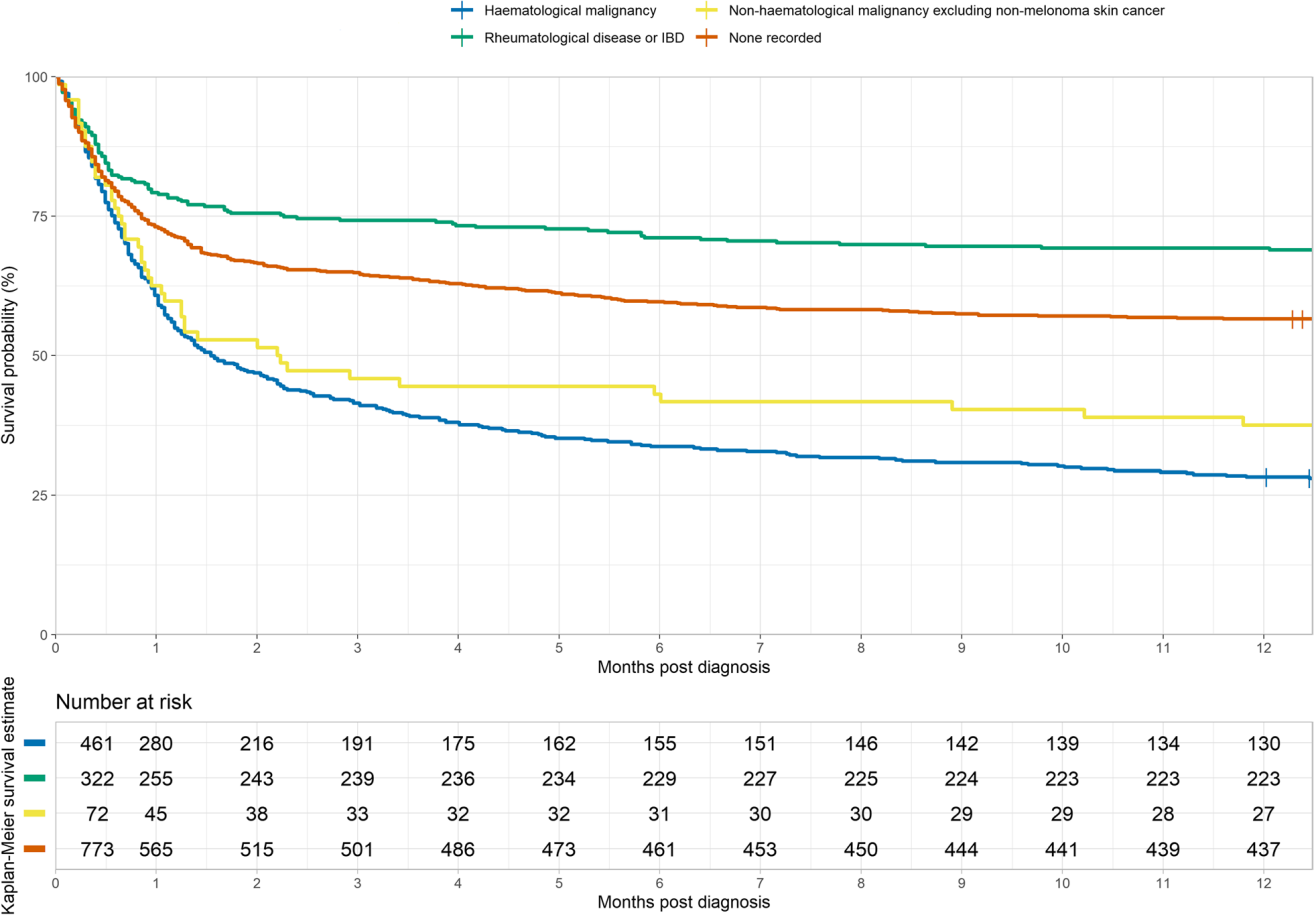
1-year survival estimates by hierarchical comorbidity

Our study provides the first estimates of survival for HLH by associated trigger factors using a population-based cohort. In terms of age, sex distribution and proportions with haematological malignancy and rheumatological diseases, the characteristics of our cohort are similar to a prior CPRD study [2] and comparable studies of HLH elsewhere [1]. The 461 patients with HLH and haematological cancer and > 500 cases of HLH in patients aged 0–14 years represent the largest respective series reported, with no comparable sized cohorts of auto-immune related HLH. Notably, patients aged 0–5 years have a greater risk of death than those 5–14 years. This is potentially due to the youngest paediatric patients having more profound genetic T- and NK-cell dysfunction, and presenting with more florid cytokine storms. Haematological malignancies complicated by HLH have dismal outcomes regardless of disease subtype, as do all patients over 55 years regardless of trigger. Potential reasons include reduced ability to tolerate acute cytokine storm and, for auto-immune disease, an assumption that cases only occur in younger people. We also critically show survival outcomes have remained static for the study-period.

## Supplementary Information

**Additional file 1.** Supplementary methods.

**Additional file 2.** Flow diagram.

**Additional file 3.** 1-year survival estimates by age.

**Additional file 4.** 1-year survival estimates by both age and co-morbidity.

**Additional file 5. Supplementary table 1.** Hazard ratios showing the interaction between age group and hierarchical co-morbidity.

**Additional file 6.** 1-year survival estimate by lymphoma subtype.

**Additional file 7.** 1-year survival estimate by non-haematological malignancy.

## Data Availability

The dataset(s) supporting the conclusions of this article is available to those that have the legal basis to access it, either through the Data Access Request Service (https://digital.nhs.uk/services/data-access-request-service-dars) or partnership with NDRS.

## Abbreviations

HLH: Haemophagocytic lymphohistiocytosis
IBD: Inflammatory bowel diseases
HES: Hospital Episode Statistics
NCRD: National Cancer Registration Dataset
ONS: Office for National Statistics
NHS: National Health Service
OPCS-4: Office of Population, Censuses and Surveys Classification of Surgical Operations and Procedures version 4
NDRS: National Disease Registration Service
CPRD: Clinical Practice Research Datalink
