# Supplementary material for: 1-year survival in haemophagocytic lymphohistiocytosis: a nationwide cohort study from England 2003–2018
Source: J Hematol Oncol. 2023 May 26;16:56. doi: 10.1186/s13045-023-01434-4 (PMC10224226; doi:10.1186/s13045-023-01434-4)
Supplement: Supplementary file 1 — Additional file 1. Supplementary methods. [file 13045_2023_1434_MOESM1_ESM.docx]

**Supplementary Methods**

Data sources

Linked electronic health records from English Hospital Episode Statistics (HES)(1), the National Cancer Registration Dataset (NCRD)(2) and Office for National Statistics (ONS) death certification data were used. In brief, HES data are collected routinely for all National Health Service (NHS) related hospital admissions in England and contain sociodemographic and clinical information, the latter coded with International Statistical Classification of Diseases and Related Health Problems, 10^th^ revision (ICD-10) and Office of Population, Censuses and Surveys Classification of Surgical Operations and Procedures version 4 (OPCS-4), NCRD contains all cancers registered in England and their associated histological and staging information and ONS death certification data contains the contents of the death certificate coded for underlying cause of death (with ICD-10), including any associated free text.

Study population and case identification

Patients diagnosed with HLH were identified using our previously validated approach(3-5). They included HLH patients of all ages who were admitted to hospital or died between 1 January, 2003 and 31 December, 2018 in England. In brief, people who had an admission coded with ICD-10 codes for HLH – D76·1 (Haemophagocytic lymphohistiocytosis) or D76·2 (Haemophagocytic syndrome) or a death coded with D76·1, D76·2 or D76·3 (Other histiocytosis syndromes) as long as in the latter situation there was confirmatory free text on the death certificate indicating HLH were included. The date of diagnosis was taken as the first day of the hospital admission in which HLH was coded. Those identified only by death registration with no preceding hospital admission were excluded, as were those without a recorded gender of male or female. Our case definition of HLH is based on a published validation exercise we carried out in five English NHS Trusts (hospitals) that showed a positive predictive value for a diagnosis of HLH of 89.0% (95% CI 80.2-94.9%)(6). In a systematic review of validation studies of other diseases, HES recording has been shown to be accurate for the purposes of research in this manner(7). Two other studies in France(8) and Chicago, USA(9) have used a similar algorithm to identify cases of HLH and NHS Digital, who oversee coding within the NHS in England, confirmed that no changes have occurred in the procedural use of D76.1, D76.2 and D76.3 during the study period. We are therefore reasonably confident that the cases we have included represent true diagnoses of HLH, and that these diagnoses are underpinned by clinical use of diagnostic scoring systems such as the “HScore”(10) or the “HLH-2004 diagnostic criteria”(11, 12). Our study design does mean that there will have been some under-ascertainment of HLH as it is recognised to be a difficult disease to diagnose(13) and therefore our capture of incidence cases is likely to be, in general, an underestimate of the true number of incident cases as cases of HLH that occurred but were not clinically diagnosed as such in the hospital setting would not have been included in our study.

HLH associated characteristics and associated comorbidities

For all patients, information on age and gender at diagnosis and determined HLH associated comorbidities was extracted. The presence or absence of comorbidities was identified from all available HES and NCRD records prior to the diagnosis of HLH and up to three months after diagnosis. Haematological cancer, non-haematological cancer (excluding non-melanoma skin cancer), inflammatory rheumatological disease, inflammatory bowel disease (IBD) were defined (supplementary tables S1, S2 and S3). For inflammatory rheumatological diseases, subcategories of diagnosis (supplementary data table S1) i.e. systemic juvenile idiopathic arthritis, systemic lupus erythematosus (SLE) etc were assigned. If more than one subcategory of inflammatory rheumatological disease was identified, the patient was assigned to the diagnosis group that was recorded closest in time to the HLH diagnosis. For the purposes of later analyses where there was overlap of non-infectious associated comorbidities, patients were classified with the following mutually exclusive hierarchy: haematological malignancy, rheumatological disease/IBD, non-haematological malignancy and none of these recorded. For patients where more than one malignancy was recorded, a panel of authors (JW, CJC, MB, PS) considered the different diagnoses and temporal relationship to the diagnosis of HLH for each case. For the sub-classification of haematological, inflammatory and other malignancies, the disease that occurred closest in time to the HLH diagnosis was assigned as the associated comorbidity. For the purposes of further survival analyses, inflammatory conditions were grouped as follows: if a patient had IBD this was assigned first then rheumatological diseases were grouped as follows: Adult onset Still’s disease (AOSD) with Systemic Juvenile Idiopathic Arthritis (SJIA); Other connective tissue disorders (OCTD) with Systemic Lupus Erythematosus (SLE); Rheumatoid arthritis together with Inflammatory Arthritis. As there was no access to serological or genetic tests for the cohort, the type (acute, chronic, reactivation) of viral illness nor genetic cause could not be ascertained.

Statistical analysis

The number of people at risk from their date of HLH diagnosis and the one-year mortality frequencies were calculated by age (categorised into 0-4, 5-14, 15-34, 35-54, 55-74, 75+ years), gender, calendar period (categorised into 3 epochs - 2003-2008, 2009-2013, 2014-2018), and the comorbidity associated with the HLH diagnosis as described above. The corresponding crude one-year survival estimates, and 95% Confidence Intervals (95% CI) were computed using Kaplan-Meier (KM) methods. Stratified KM survival curves to show survival over the year following HLH diagnosis by age category and by the comorbidity associated with the HLH diagnosis were then plotted.

A Cox regression model was fitted, adjusted by age, gender, co-morbidities and calendar time period to assess if any observed differences in survival were confounded. An interaction between age and co-morbidity was fitted and tested with a general likelihood ratio test to assess if age related mortality varied by the associated co-morbidity. Finally, to assess if mortality trends over the study period varied by age and co-morbidity, we fitted interactions between age and calendar year, and between co-morbidity and calendar year, and tested them with general likelihood ratio tests. Where appropriate, when statistically significant interactions were observed, we calculated computed stratum-specific adjusted HRs and 95% CIs for one-year survival.

Study findings are reported in accordance with the REporting of studies Conducted using Observational Routinely collected health Data (RECORD) recommendations(14). We used R (version 4·1·2)(15) for the data management and statistical analyses.

1. Herbert A, Wijlaars L, Zylbersztejn A, Cromwell D, Hardelid P. Data Resource Profile: Hospital Episode Statistics Admitted Patient Care (HES APC). Int J Epidemiol. 2017;46(4):1093-i.

2. Henson KE, Elliss-Brookes L, Coupland VH, Payne E, Vernon S, Rous B, et al. Data Resource Profile: National Cancer Registration Dataset in England. Int J Epidemiol. 2020;49(1):16-h.

3. West J, Stilwell P, Liu H, Ban L, Bythell M, Card TR, et al. Temporal trends in the incidence of haemophagocytic lymphohistiocytosis: a nationwide cohort study from England 2003-2018. medRxiv. 2022:2022.03.30.22273090.

4. West J, Card TR, Bishton MJ, Lanyon P, Ban L, Bythell M, et al. Incidence and survival of haemophagocytic lymphohistiocytosis: A population-based cohort study from England. J Intern Med. 2022;291(4):493-504.

5. Bishton MJ, Stilwell P, Card TR, Lanyon P, Ban L, Elliss-Brookes L, et al. A validation study of the identification of haemophagocytic lymphohistiocytosis in England using population-based health data. Br J Haematol. 2021;194(6):1039-44.

6. Bishton M, Stilwell P, Card TR, Lanyon P, Ban L, Elliss-Brookes L, et al. A validation study of the identification of haemophagocytic lymphohistiocytosis in England using population-based health data. medRxiv. 2021:2021.05.18.21257169.

7. Burns EM, Rigby E, Mamidanna R, Bottle A, Aylin P, Ziprin P, et al. Systematic review of discharge coding accuracy. J Public Health (Oxf). 2012;34(1):138-48.

8. Riviere S, Galicier L, Coppo P, Marzac C, Aumont C, Lambotte O, et al. Reactive hemophagocytic syndrome in adults: a retrospective analysis of 162 patients. Am J Med. 2014;127(11):1118-25.

9. Jumic S, Nand S. Hemophagocytic Lymphohistiocytosis in Adults: Associated Diagnoses and Outcomes, a Ten-Year Experience at a Single Institution. J Hematol. 2019;8(4):149-54.

10. Fardet L, Galicier L, Lambotte O, Marzac C, Aumont C, Chahwan D, et al. Development and validation of the HScore, a score for the diagnosis of reactive hemophagocytic syndrome. Arthritis Rheumatol. 2014;66(9):2613-20.

11. La Rosee P, Horne A, Hines M, von Bahr Greenwood T, Machowicz R, Berliner N, et al. Recommendations for the management of hemophagocytic lymphohistiocytosis in adults. Blood. 2019;133(23):2465-77.

12. Henter JI, Horne A, Arico M, Egeler RM, Filipovich AH, Imashuku S, et al. HLH-2004: Diagnostic and therapeutic guidelines for hemophagocytic lymphohistiocytosis. Pediatr Blood Cancer. 2007;48(2):124-31.

13. Ramos-Casals M, Brito-Zeron P, Lopez-Guillermo A, Khamashta MA, Bosch X. Adult haemophagocytic syndrome. Lancet. 2014;383(9927):1503-16.

14. Benchimol EI, Smeeth L, Guttmann A, Harron K, Moher D, Petersen I, et al. The REporting of studies Conducted using Observational Routinely-collected health Data (RECORD) statement. PLoS Med. 2015;12(10):e1001885.

15. Team RC. R: A Language and Environment for Statistical Computing. Vienna, Austria: R Foundation for Statistical Computing; 2022 2022.
