# Supplementary figures and images for: 1-year survival in haemophagocytic lymphohistiocytosis: a nationwide cohort study from England 2003–2018
Source: J Hematol Oncol. 2023 May 26;16:56. doi: 10.1186/s13045-023-01434-4 (PMC10224226; doi:10.1186/s13045-023-01434-4)

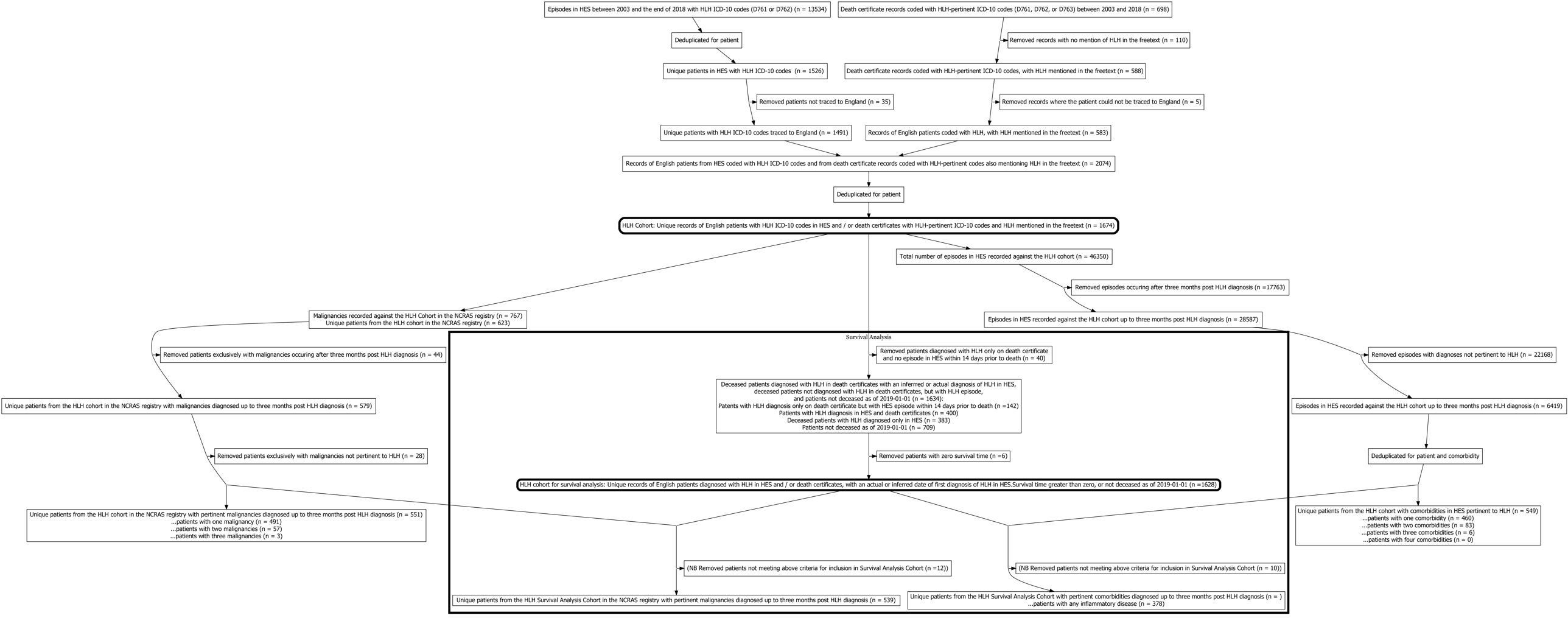

Supplement: Supplementary file 2 — Additional file 2. Flow diagram. [file 13045_2023_1434_MOESM2_ESM.pdf]

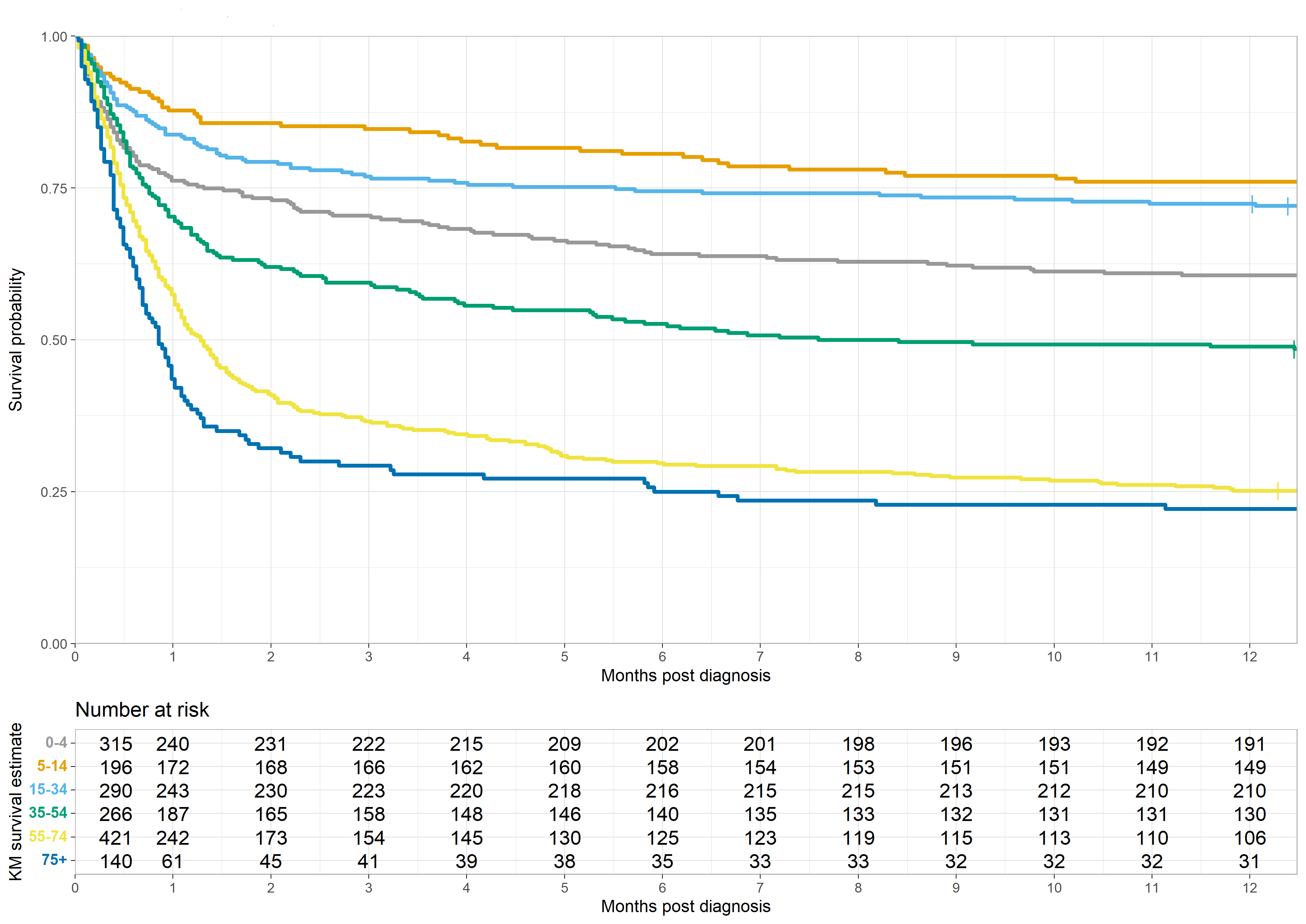

Supplement: Supplementary file 3 — Additional file 3. 1-year survival estimates by age. [file 13045_2023_1434_MOESM3_ESM.tiff]

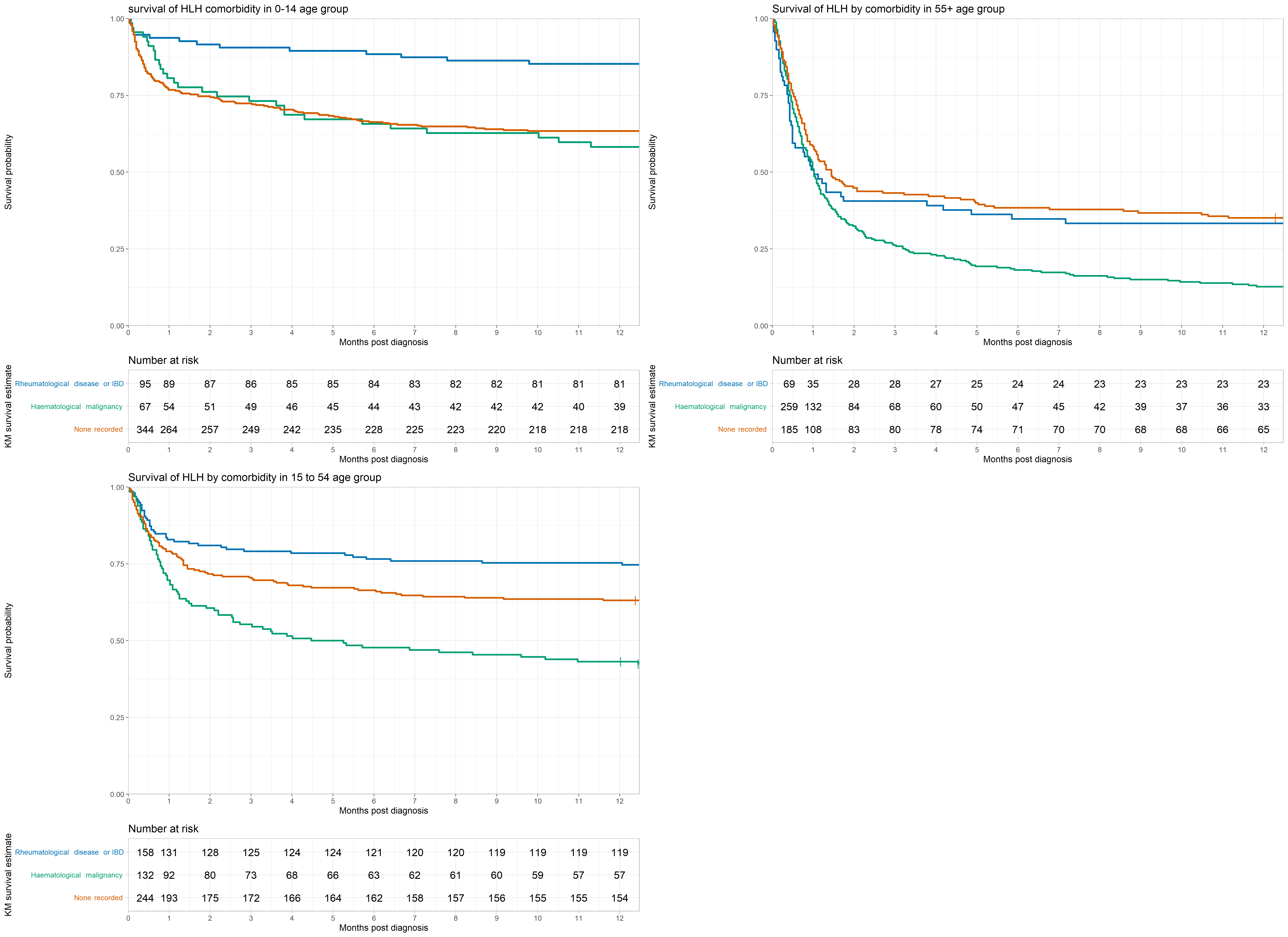

Supplement: Supplementary file 4 — Additional file 4. 1-year survival estimates by both age and co-morbidity. [file 13045_2023_1434_MOESM4_ESM.tiff]

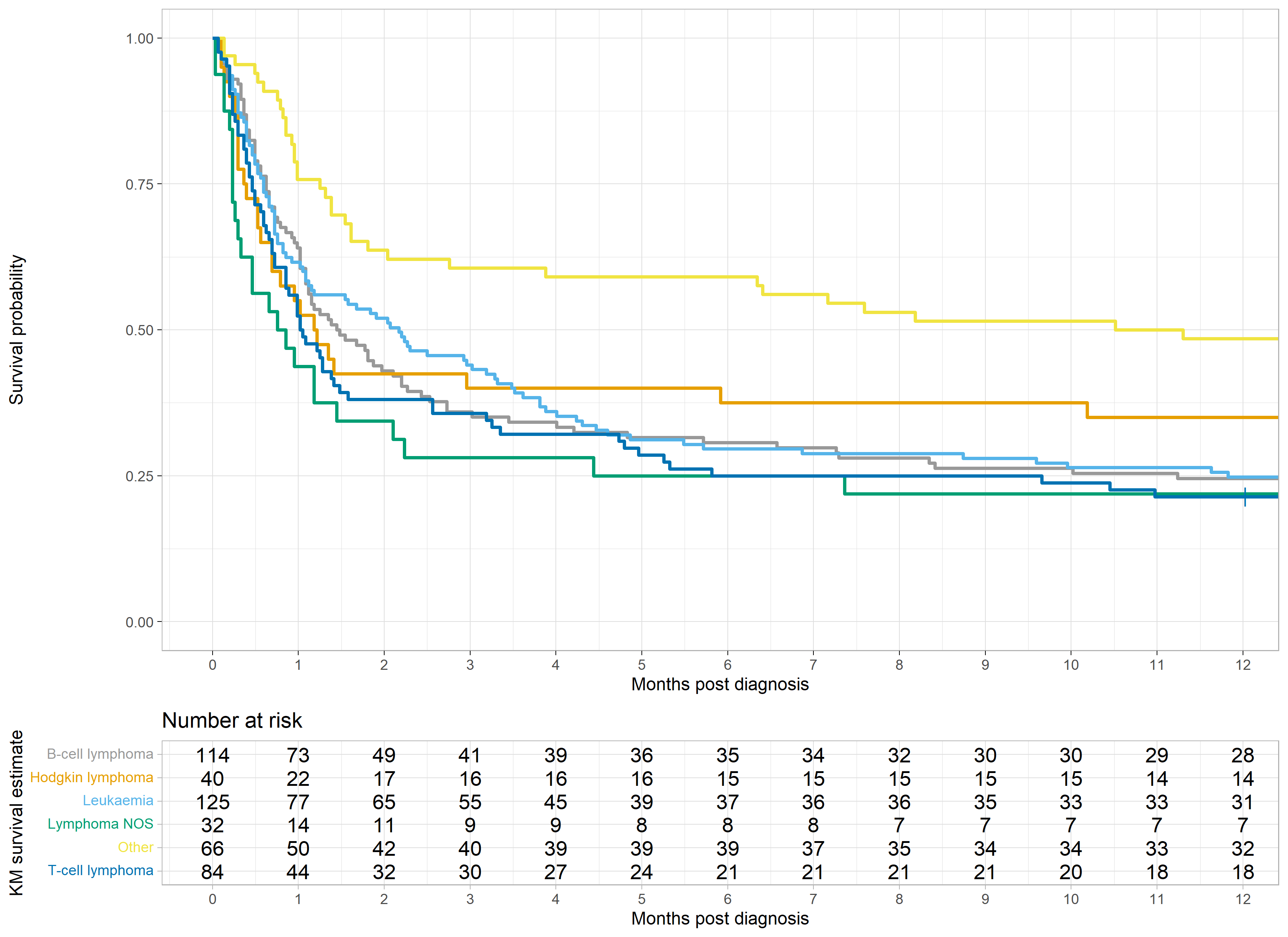

Supplement: Supplementary file 6 — Additional file 6. 1-year survival estimate by lymphoma subtype. [file 13045_2023_1434_MOESM6_ESM.tiff]

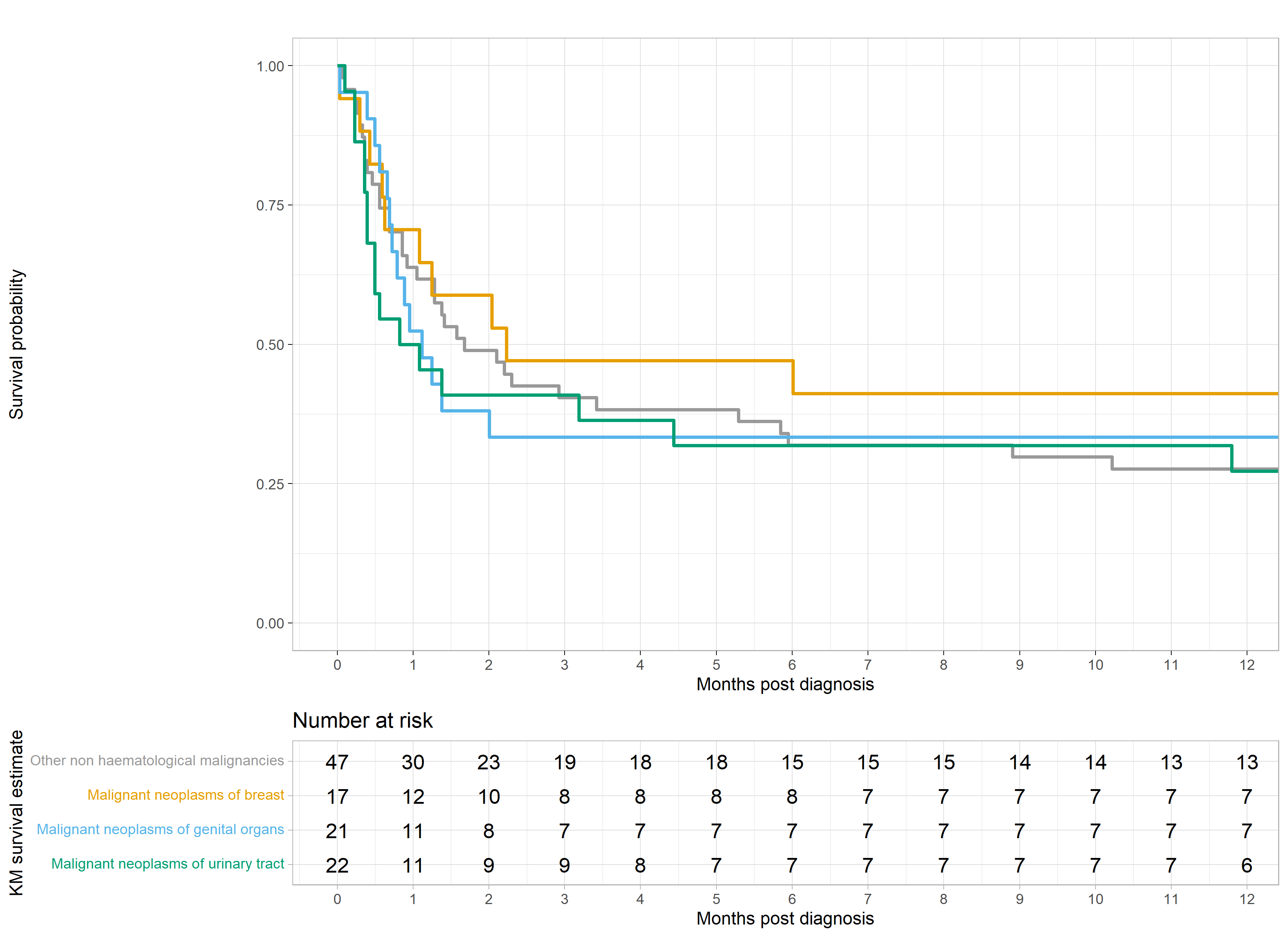

Supplement: Supplementary file 7 — Additional file 7. 1-year survival estimate by non-haematological malignancy. [file 13045_2023_1434_MOESM7_ESM.tiff]
