# Supplementary material for: 1-year survival in haemophagocytic lymphohistiocytosis: a nationwide cohort study from England 2003–2018
Source: J Hematol Oncol. 2023 May 26;16:56. doi: 10.1186/s13045-023-01434-4 (PMC10224226; doi:10.1186/s13045-023-01434-4)
Supplement: Supplementary file 5 — Additional file 5. Supplementary table 1. Hazard ratios showing the interaction between age group and hierarchical co-morbidity. [file 13045_2023_1434_MOESM5_ESM.docx]

Supplementary table 1. Hazard ratios showing the interaction between age group and hierarchical comorbidity group

| Age | Hierarchical comorbidity | Predicted survival at 1 year^*^ | Predicted survival at 1 year CI (95%)^†^ | Hazard ratio^‡^ | Hazard ratio CI (95%)^†^ |
| --- | --- | --- | --- | --- | --- |
| 0-14 | Rheumatological disease or IBD | 0·84 | (0.75 to 0.90) | 1·00 | - |
|  | Haematological malignancy | 0·58 | (0.45 to 0.69) | 3·21 | (1.31 to 7.85) |
|  | Non-haematological malignancy excluding non-melanoma skin cancer | 0·48 | (0.13 to 0.76) | 4·31 | (0.76 to 24.45) |
|  | None recorded | 0·60 | (0.53 to 0.67) | 2·96 | (1.37 to 6.41) |
| 15-54 | Rheumatological disease or IBD | 0·73 | (0.65 to 0.80) | 1·00 | - |
|  | Haematological malignancy | 0·43 | (0.34 to 0.51) | 2·73 | (1.59 to 4.70) |
|  | Non-haematological malignancy excluding non-melanoma skin cancer | 0·41 | (0.20 to 0.61) | 2·87 | (1.17 to 7.07) |
|  | None recorded | 0·60 | (0.52 to 0.67) | 1·62 | (0.96 to 2.74) |
| 55+ | Rheumatological disease or IBD | 0·27 | (0.17 to 0.38) | 1·00 | - |
|  | Haematological malignancy | 0·16 | (0.12 to 0.21) | 1·39 | (0.89 to 2.16) |
|  | Non-haematological malignancy excluding non-melanoma skin cancer | 0·31 | (0.19 to 0.44) | 0·89 | (0.47 to 1.67) |
|  | None recorded | 0·32 | (0.25 to 0.39) | 0·87 | (0.54 to 1.40) |
| ^*^ 95% Confidence Interval |  |  |  |  |  |
| ^†^ Predictions set for males in 2018 | ‡ Within age-group hazard ratios for the hierarchical comorbidities are fully adjusted for gender and the year of diagnosis. Age by hierarchical comorbidity interaction P = 0.001 |  |  |  |  |
|  |  |  |  |  |  |
